# Masked mycotoxins: A review

**DOI:** 10.1002/mnfr.201100764

**Published:** 2012-10-10

**Authors:** Franz Berthiller, Colin Crews, Chiara Dall'Asta, Sarah De Saeger, Geert Haesaert, Petr Karlovsky, Isabelle P Oswald, Walburga Seefelder, Gerrit Speijers, Joerg Stroka

**Affiliations:** 1 Christian Doppler Laboratory for Mycotoxin Metabolism, Department for Agrobiotechnology (IFA-Tulln), University of Natural Resources and Life Sciences ViennaTulln, Austria; 2 Food and Environment Research AgencyYork, UK; 3 Department of Organic and Industrial Chemistry, University of ParmaParma, Italy; 4 Laboratory of Food Analysis, Department of Bioanalysis, Ghent UniversityGhent, Belgium; 5 Faculty of Applied Bio-Engineering, University College of GhentGhent, Belgium; 6 Molecular Phytopathology and Mycotoxin Research Section, University of GoettingenGoettingen, Germany; 7 INRA, UMR 1331 ToxAlim, Research Center in Food ToxicologyToulouse, France; 8 Quality and Safety Assurance Department, Nestlé Research Center, Nestec Ltd.Lausanne, Switzerland; 9 General Health Effects Toxicology Safety Food (GETS)Nieuwegein, The Netherlands; 10 Institute for Reference Materials and Measurements (IRMM), European Commission Joint Research CentreGeel, Belgium

**Keywords:** Bound Mycotoxins, Conjugated Mycotoxins, Masked Mycotoxins, Plant Metabolism

## Abstract

The aim of this review is to give a comprehensive overview of the current knowledge on plant metabolites of mycotoxins, also called masked mycotoxins. Mycotoxins are secondary fungal metabolites, toxic to human and animals. Toxigenic fungi often grow on edible plants, thus contaminating food and feed. Plants, as living organisms, can alter the chemical structure of mycotoxins as part of their defence against xenobiotics. The extractable conjugated or non-extractable bound mycotoxins formed remain present in the plant tissue but are currently neither routinely screened for in food nor regulated by legislation, thus they may be considered masked. *Fusarium* mycotoxins (deoxynivalenol, zearalenone, fumonisins, nivalenol, fusarenon-X, T-2 toxin, HT-2 toxin, fusaric acid) are prone to metabolisation or binding by plants, but transformation of other mycotoxins by plants (ochratoxin A, patulin, destruxins) has also been described. Toxicological data are scarce, but several studies highlight the potential threat to consumer safety from these substances. In particular, the possible hydrolysis of masked mycotoxins back to their toxic parents during mammalian digestion raises concerns. Dedicated chapters of this article address plant metabolism as well as the occurrence of masked mycotoxins in food, analytical aspects for their determination, toxicology and their impact on stakeholders.

## 1 Introduction

Health hazards from food can be caused by infectious agents and toxic compounds [1, 2]. Living microorganisms ingested with food can cause infectious diseases, while toxic substances lead to acute poisoning or have a long-term negative impact on the health of consumers. Chemical food contaminants originate mainly from the following sources: unintentional pollutants, intentionally added compounds at levels exceeding legal limits or in commodities for which they have not been approved, toxic plant metabolites, contaminants generated by processing and toxic microbial metabolites. While a general assessment of the impact of these contaminants on public health is difficult to make, it can be argued that direct measures are in place to curb the impact of man-made contaminants. Obligatory approval for synthetic compounds entering the food chain, such as pesticides and preservatives include their toxicological assessment, and guidelines for the application. These safeguards cannot be applied to naturally occurring toxins. Most importantly, only indirect control of the level of natural toxins in food commodities by measures such as good manufacturing practices, soil treatment, the use of resistant varieties and fungicide application is possible [3]. For these reasons, the threat to the health of the consumer posed by natural toxicants appears more serious than the health risk posed by man-made pesticides, preservatives and other food additives [4].

Natural toxins in food are plant secondary metabolites, bacterial toxins, phycotoxins and mycotoxins. Mycotoxins are secondary metabolites of fungi toxic to animals and humans, and have been reviewed (e.g. [5]). Fungi that produce mycotoxins relevant to agriculture are phytopathogenic organisms that infect living plants in the field and/or greenhouse and saprophytic fungi that colonise plant products post harvest [6]. While only a small number of plant pathogenic fungal species are known to produce mycotoxins, most spoilage fungi secrete a range of toxic metabolites. The most important fungal genera producing mycotoxins that are found in food products are *Aspergillus, Fusarium, Alternaria* and *Penicillium*.

Mycotoxin derivatives that are undetectable by conventional analytical techniques because their structure has been changed in the plant are designated masked mycotoxins [7, 53]. In the following, the term conventional analytical detection addresses those methods that have initially been developed for specific mycotoxins only. It must however be stressed that some conventional methods such as ELISA, might also respond to masked forms, whereas this is unlikely for HPLC-based methods. Chemical transformations that generate masked mycotoxins are catalyzed by plant enzymes, most commonly by enzymes involved in detoxification processes.

Food processing, can on the other hand also chemically alter mycotoxins, however most of the food-processing compounds are less toxic than their precursors. Microorganisms used in fermentation processes may transform mycotoxins into products that are also not detected by analytical methods conventionally used for mycotoxin monitoring. These derivatives resulting from the enzymatic activities of microbial cultures used for fermentation, such as in the manufacturing of wine, beer, fermented sausages or mixed pickles, have so far not been studied.

The group of masked mycotoxins comprises both extractable conjugated and bound (non-extractable) varieties. Bound mycotoxins are covalently or non-covalently attached to polymeric carbohydrate or protein matrices [8]. Extractable conjugated mycotoxins can be detected by appropriate analytical methods when their structure is known and analytical standards are available. Bound mycotoxins, however, are not directly accessible and have to be liberated from the matrix by chemical or enzymatic treatment prior to chemical analysis.

The definition of masked mycotoxins implies that the analysis of the mycotoxin content of samples containing these compounds leads to their underestimation. Masked mycotoxins may elude analysis because of changed physicochemical properties of their molecules leading to modified chromatographic behaviour, because of modification of an epitope recognised by antibodies used for the detection, or because of impaired extraction efficiency caused by increased polarity when a less polar solvent is used for the extraction of non-modified mycotoxins. Bound mycotoxins completely elude conventional analysis. All of these effects lead to underestimation of the total mycotoxin content of the sample. Modifications of mycotoxin molecules that reduce or eliminate toxicity, on the other hand, may lead to apparent overestimation of mycotoxin contamination. This happens when the analytical method detects the modified mycotoxin along with the unmodified molecule but does not reveal that the analytical signal originated from a less toxic or non-toxic derivative. This is particularly relevant for methods based on antigen–antibody binding because epitopes recognised by antibodies and toxicity determinants destroyed by the modification are not necessary identical.

Some transformations that generate masked mycotoxins may lead to a decrease of toxicity. These processes should be designated detoxification rather than masking, as, except for scientific curiosity, the detection of non-toxic forms of mycotoxins in food products is not required. Bound mycotoxins may be regarded as detoxified as long as they cannot be released from the matrix during food processing or in the digestive system. Classification of mycotoxin transformations as masking or detoxification is therefore only possible when the fate of the substances during food processing and digestion is understood. Toxicity assessment for all mycotoxin derivatives that occur in food is important for the estimation of the health risk posed by the sum of different forms of a given mycotoxin. It should be a high priority for research to extend current multitoxin methods to include newly discovered transformation products of mycotoxins. Although the task is technically feasible, continuous extension of analytical methods and timely adoption of the modified methods on a large scale across countries and districts is likely to face significant administrative, financial and organisational hurdles. Monitoring of zearalenone (ZEN) in feeds illustrates this problem: the risk of hyperestrogenic effects is underestimated because α-zearalenol (α-ZEL), which is a more estrogenic derivative of ZEN, is neither often determined nor regulated. The recognition of the toxicological relevance of masked mycotoxins in food commodities provides a new impetus for the establishment of overall toxicity estimates to be used by regulatory bodies, food manufacturers and monitoring authorities to protect consumers’ health.

The aim of this review is to summarise the current knowledge of the determination, occurrence, toxicity and impact of masked mycotoxins.

## 2 Plant metabolism related to masked mycotoxins

### 2.1 Plant detoxification systems

Plants have versatile detoxification systems to counter a wide variety of non-natural as well as natural phytotoxic chemical compounds. Among these compounds, mycotoxins are a target of the plants’ detoxification metabolic processes since they can interact with vital cell functions. Plants are endowed with two major detoxification mechanisms: chemical modification and compartmentation.

Two types of reactions are responsible for the chemical modifications of xenobiotics in animals. Phase I reactions usually involve hydrolysis or oxidation while phase II reactions are characterised by conjugation. Chemical transformations in phase I are typical for lipophilic xenobiotics, which means that most of the hydrophilic toxic compounds are not affected by this phase. Hydrolysis in phase I is catalyzed by esterases and amidases, but oxidations catalyzed by the cytochrome P-450 system are the most prevailing reactions [9, 10]. The reactions in phase I do not always lead to components with decreased phytotoxicity compared to the original xenobiotic itself; in some cases, the metabolite is as toxic as the parent compound, and in others, there is even a considerable increase in toxicity [9].

Phase II enzymes deactivate phase I activated metabolites or xenobiotics in a direct way by covalent binding of hydrophilic molecules. Plants have evolved a battery of sophisticated conjugation reactions: glucose, malonic acid and glutathione (GSH, γ-glutamyl-cysteinyl-glycine) are all residues that can bind to functional groups of xenobiotics. Glucosyl residues conjugate with hydroxy, thiol, amino and carboxy groups while malonyl residues conjugate with hydroxy and amino groups. Finally, GSH residues have an affinity with electrophilic sites in the molecule. These phase II reactions are catalyzed by glucosyl-, malonyl- and glutathione-*S*-transferases (GSTs), respectively. Unlike phase I reactions, which can produce phytotoxic metabolites, phase II products are either non-toxic or less toxic than the parent compound [9]. The transfer of hydrophilic groups by the phase II metabolism changes the chemical properties of the xenobiotics, makes them more water soluble, alters their bioactivity and often enables access to membrane transporter systems [11]. Finally in plants, the phase II modification leads to elimination of the toxic components from the cytosol via membrane-bound transporters into the vacuolar or apoplastic space [9, 11]. The carriers used for this transport to the tonoplast or the plasmamembrane are most likely different for glycosylated, malonylated and glutathionylated xenobiotics, respectively. An increasing amount of evidence suggests that glycosylated conjugates use a carrier system energetically coupled to the transmembrane H^+^ gradient (P type ATPase) for transport across the tonoplast while glutathionylated forms are transported by ABC transporter(s) directly fuelled by adenosine-5′-triphosphate (ATP) [12].

There are many studies linking partial detoxification of exogenously administered chemicals with UDP-glucosyltransferase (UGT) activities in planta [13–15]. UGTs of family 1 in particular are involved in the detoxification of xenobiotics. Studies based on recombinant expression of glucosyl transferases in *Arabidopsis thaliana* exhibit clear in vitro activities towards a variety of endogenous plant compounds [16]. Data suggest that there is substrate specificity of UGTs in relation to the glycosylation process of chemical groups, although information derived from experiments with knock out mutants shows that different UGTs could compensate each other for glycosylation. Competition studies have shown that certain xenobiotics (e.g. 2,4,5 trichlorophenol) affect the activities of UGTs towards naturally occurring substrates and vice versa, suggesting that cross-talk between detoxification of xenobiotics and endogenous metabolites may occur in plants, depending on the presence of UGT competing substrates [11, 17].

Apart from glycosylation, conjugation with GSH is the most important phase II detoxification mechanism in plants [18]. Different electrophilic centres on mycotoxin molecules can be attacked by GSH in reactions catalyzed by GSTs, which are enzymes with wide substrate specificity. In its role as a nucleophilic ‘scavenger’, GSH can undergo spontaneous or GST-catalyzed conjugation to a wide range of xenobiotic electrophiles [9]. Unlike animal GSTs, the active centre of plant GSTs possesses a serine residue. Conjugation with GSH attaches a side group containing two carboxyls, an amine group, two peptide bonds and a thiol, which renders this part of the molecule highly polar and hydrophilic. An important consequence of conjugation with GSH is that the conjugates cannot cross-biological membranes and move freely among compartments. At physiological pH values, the GSH residue is subject to ionisation that prevents diffusion across phospholipid bilayers. Specific transporters are necessary for the transfer of such conjugates, e.g. from cytoplasm to the vacuole. Another distinguishing feature of GSH conjugation is the irreversibility of the reaction. Degradation of GSH conjugates is possible but the products are different from the original xenobiotic. The only potential exception is addition of GSH to a double bond next to an electron withdrawing group. Conjugations with epoxides, lactones or aldehyde groups, which are more relevant for mycotoxins, are likely to be irreversible. This situation is very different from the conjugation of mycotoxins to glucose, which can be reversed by numerous glycosidases present in plants and/or in the digestive system of animals. While most conjugates generated by GSTs in plants contain GSH, some members of the plant GST super family accept GSH derivatives, generating conjugates with homoglutathione and hydroxymethylglutathione [19]. This is important to consider when developing analytical methods for masked mycotoxins conjugated with GSH. The activity of GSTs is induced by stress, e.g. by heavy metals [20]. This phenomenon is the mode of action of herbicide safeners that induce GST activity in crops [21]. It can therefore be assumed that the level of mycotoxins masked by GSH conjugation in plants varies to a large degree and that herbicide treatments stimulate the conversion of mycotoxins to masked forms. Conjugation with GSH is an important detoxification reaction for aflatoxins in animals [22]. Conjugation of trichothecenes with GSH in plants was indicated already in the 1970s [23], optimisation of GST towards deoxynivalenol (DON) was patented by Maxygen in 2002 [24]. Though the extent of the conjugation of trichothecenes with GSH in planta is not known, recent results showed that the reaction occurs in vitro [25].

### 2.2 Plant versus animal mycotoxin metabolism

Although there are only limited data describing the metabolism of mycotoxins in plants and animals, some comparison is possible. In general, plants can metabolise xenobiotic compounds including mycotoxins as part of their defence against a pathogens. From metabolism studies in plants it is known that, as in animals, distinctions can be made between the metabolism in a phase I process (enzymatic transformation such as reduction, oxidation or hydrolysis) and a phase II process (conjugation such as glucosidation, glucuronidation or sulfatation) [8, 9, 26, 27]. While both processes aim to detoxify the xenobiotic mycotoxin, phase I transformation processes may as well lead to activation and thus to a higher toxicity. In phase II, conjugation reactions lead to the formation of more water soluble (hydrophilic) compounds facilitating the elimination of the mycotoxin, thus decreasing the toxicity. The metabolism in different food plants is similar in a qualitative manner, however, there can be some quantitative difference between the different food plants [9]. This implies that metabolic data for a food plant can only be extrapolated to other food plants qualitatively. While there is some similarity with mycotoxin metabolism in animals, here the difference between plants and animals lies within distribution and elimination. In plants, compartmentalisation plays an important role, but in animals there is active elimination (excretion), usually involving the kidney and liver. It can be stated that the similarity between plants and animals is particularly clear with respect to conjugation reactions.

Phase III detoxification reactions in plants involve sequestration of compounds conjugated to glucose or GSH into the vacuole or their irreversible binding to the cell wall. In this way, detoxification products are permanently stored in the plant tissue rather than excreted. The only mechanism that allows plants to excrete detoxified metabolites efficiently into the environment is root exudation. It is unlikely that fungal toxins produced in shoots would be transformed to the roots and exuded, though a long-range transport of a particular GSH conjugate into the roots and its secretion by root tips has been described [28]. The majority of toxins conjugated with GSH are found in the vacuole. There the conjugates may be subject to further transformations. For example, GSH conjugates may undergo hydrolysis of the peptide bond of GSH, leading to γ-glutamylcysteinyl-S-conjugates [29]. Numerous further transformations of GSH conjugates have been observed [30] but it is not known whether these processes occur with mycotoxin conjugates.

### 2.3 Plant breeding

Pursuing a genetic approach, Lemmens and coworkers demonstrated that the ability of wheat lines to convert DON to deoxynivalenol-3-β-d-glucopyranoside (D3G) was linked to a quantitative trait locus (QTL), designated Qfhs.ndsu-3BS which had been previously reported to be associated with Fusarium head blight (FHB) resistance^31^. This study provided the first lines of evidence for a link between resistance to the FHB pathogen and the ability of plants to metabolise the mycotoxins of this pathogen. The presence of the resistance gene *Fhb1* linked to Qfhs.ndsu-3BS clearly decreases FHB symptoms but raises the D3G/DON ratio. Nevertheless, *Fhb1* reduces the sum of parent and masked DON significantly. Pyramiding of more QTL's for FHB resistance shows a positive additive effect on plant resistance and DON accumulation^32^.

To date, numerous candidate UGT genes with a possible role in DON detoxification have been identified in wheat and barley, based on their increased activity upon a *Fusarium* infection, a DON treatment or a different expression profile in varieties with differential FHB resistance. All these candidate UGT genes encode for enzymes transferring glucose to small molecules [33]. Using the Affymetrix GeneChip technology nine and six UGT genes have been shown to be upregulated during a *Fusarium* infection in barley and wheat, respectively [34, 35]. Research based on four barley UGT genes and a wheat UGT gene indicated that only one of the proposed barley genes serves as a DON-glucosyltransferase leading to DON resistance and that the proposed wheat gene (TaUGT3) was inactive [33]. Therefore, validation of a proposed function of a candidate UGT gene is highly recommended before investing resources into breeding efforts.

## 3 Occurrence of masked mycotoxins in plant-based food and feed

**Figure 1:**
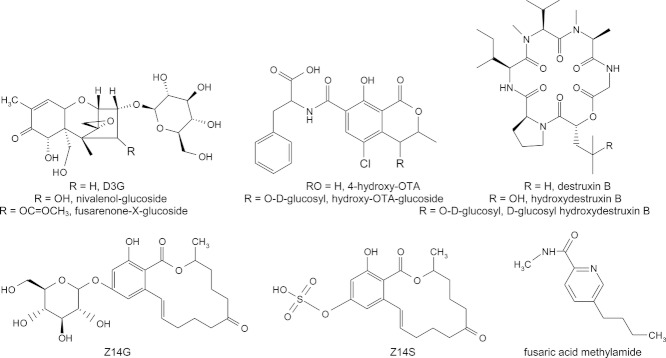
Structurally elucidated masked mycotoxins.

Plant metabolites have been identified so far for DON, nivalenol, fusarenon-X, T-2 toxin, HT-2 toxin, ZEN, ochratoxin A (OTA), destruxins and fusaric acid (Fig. 1). Moreover, there is some evidence for the compartmentation of fumonisins in plants. Generally, cell cultures have been used for the isolation and structural identification of mycotoxin metabolites. Up to now, only zearalenone-14-β-D-glucopyranoside (Z14G) and D3G have been proven to occur in naturally infected cereals such as wheat, barley and maize, while fusaric acid methylamide was shown to occur in infected vegetables. While the occurrence of bound (also called hidden) fumonisins in raw maize as well as in cereal derived food has been proven, the nature of the masking mechanism has not been completely clarified.

### 3.1 Masked trichothecenes

It is known that cereal crops infected with DON-producing fungi are capable of detoxifying this mycotoxin. In this context, one major pathway is the conjugation of DON to a glucose moiety giving rise to D3G, which has been isolated from *Zea mays* suspension cultures that were treated with DON [36] and from contaminated wheat [37]. D3G exhibited a dramatically reduced ability to inhibit protein synthesis of wheat ribosomes in vitro [38]. Miller and coworkers first speculated that formation of a less toxic DON conjugate might be responsible for partial FHB resistance of wheat [39]. Furthermore, a UDP-glucosyl transferase from *A. thaliana* has been identified, which catalyzes the transfer of glucose from UDP-glucose to the hydroxyl group at the carbon 3 of DON [38].

The acetylated derivatives of DON, 3-acetyl-DON (3ADON) and 15-acetyl-DON (15ADON), which have been generally reported to occur together with DON especially in cereal commodities are usually considered as fungal derived metabolites [40]. The implementation of a 3-O-acetyltransferase converting DON to 3ADON has been reported for transgenic rice, wheat and barley (reviewed in [41]). However, no varieties exhibiting this trait have yet been commercialised.

So far, D3G has been described as occurring in wheat (grains, semolina and flour) [37, 42], maize (grains) [e.g. [43], oats (flour) [44], barley, malt and beer [45, 46]. Two publications [43, 44] have presented data on cereal commodities showing that the relative proportion of D3G to DON is rather stable, in average at around 20% in the 77 + 22 = 99 cereal samples considered. However, the D3G/DON ratio varied in relation to years and genotypes but reached levels up to 46% [43] and even 70% [47]. Kostelanska and coworkers found D3G levels in beer that exceed the DON concentration [46]. In a survey on extractable conjugated *Fusarium* mycotoxins in cereal raw materials and cereal-based finished products (e.g. bread, snacks, biscuits, pasta, infant food and beer), D3G was detected in two of the 84 samples analysed [48]. D3G was detected in wheat bread and in wholemeal wheat bread, but the levels were below the LOQ (100 μg/kg). Using a more sensitive method, 80% of 116 flour, breakfast cereal and snack samples from the Czech market analysed were found to be contaminated with D3G at concentrations ranging from 5 to 72 μg/kg [49]. Interestingly, Sasanya and coworkers reported that some wheat samples contained significantly higher values (up to 2.7-fold) of D3G compared to DON [42]. Furthermore, D3G was included in a Chinese survey on corn and wheat samples [50]. Median levels of 21 μg/kg D3G for wheat (100 positive of 192 samples, range: 2–238 μg/kg) and 35 μg/kg D3G for corn (69 positive of 204 samples; range 2–499 μg/kg) were reported. The median levels for DON in the same survey were 31 μg/kg for wheat (169 positive of 192 samples; range 2–591 μg/kg) and 95 μg/kg for corn (103 positive of 204 samples; range 2–4374 μg/kg), respectively. The data as presented allowed the conclusion that for some samples D3G must have exceeded the amounts of DON. One hundred fifty durum wheat samples harvested in Italy in 2010 were analysed for DON and D3G. DON was found in all of the samples at levels ranging between 0.05 and 3.74 mg/kg (mean value: 1.37 mg/kg); D3G was found in 130 of 150 samples ranging between 0.05 and 0.85 mg/kg (mean value: 0.30 mg/kg) (Dall'Asta et al., unpublished data).

There are indications that D3G can be released during food processing as a consequence of enzymatic degradation of polysaccharides. For example, it has been shown that D3G levels increase after malting of barley grains and the contaminant is transferred into beer. Whereas levels of D3G were below the LOD in the grains, their levels increased during the germination process leading to an accumulation of the DON conjugate in the germ bud. This ‘waste product’ of the malting process is used by feed industry and food supplement providers because of its high protein content [45]. Some preliminary data have also suggested the presence of di- and tri-glycosylated forms of DON in beer that are considered to be degradation products of oligo-glycosylated precursors [51]. However, DON attached to more than one sugar moiety has not been systematically analysed so far and therefore its contribution to the overall DON content is unclear. Preliminary data in this context were collected after quantification of masked DON in naturally infected barley samples by applying an acidic hydrolysis procedure [52]. In the 18 samples analysed, masked DON was present at 6 to 21% of the free DON. Furthermore, an acidic hydrolysis procedure was applied on corn and wheat samples to determine the presence of masked DON by ELISA [53]. An increase of 7–75% of DON was seen in corn and wheat following the hydrolysis procedure. This increase could not be explained by the presence of 3ADON and 15ADON. In a follow-up survey, 72 of 86 corn samples were analysed positive for masked DON [54]. The average increase of DON after trifluoromethanesulfonic acid (TFMSA) hydrolysis was 14%.

A new *Fusarium* mycotoxin glucoside, fusarenon-X-glucoside, has recently been reported for the first time in wheat grain that was artificially infected with *Fusarium* fungi. Another mycotoxin glucoside, nivalenol-glucoside was also found in the same grain sample. The authors estimated that more than 15% of fusarenon-X and nivalenol were converted into their respective glucosides [55].

3-*O*-glucosides of T-2 toxin and HT-2 toxinhave also been discovered in wheat and oats inoculated with *Fusarium sporotrichiodes*, strongly suggesting the natural occurrence of these compounds as well [56].

Glucosides of macrocyclic trichothecenes (e.g. verrucarin-A-glucoside, roridine A, D, and E glucoside) were found in the poisonous plant *Baccharis coridifolia*, which might obtain the trichothecene precursors from plant-associated fungi [57, 58]. It remains unclear, however, whether the glucosides are less toxic storage forms and part of the mechanism of self-protection of the plant. The release of the toxic aglycons by glucosidases after exposure to herbivores might be required for animal toxicity [8].

Some studies have reported the possibility of acyl conjugation of mycotoxins in plants. The synthesis of these types of conjugates could be catalyzed by acyltransferases. Acyl conjugates such as palmitoyl trichothecolone, palmitoyl scirpentriol and palmitoyl T-2 have been described after a natural and artificial infection of banana with *F. verticillioides* (syn. *F. moniliforme*) [59] but the results have been refuted by others [60]. Another example is the cinnamic acid ester of trichothecolone in anise seeds *infected with Trichothecium roseum* [61].

### 3.2 Masked ZEN

ZEN, an estrogenic compound produced by several *Fusarium* species during infection of small-grain cereals and maize, is transformed efficiently to its glucose conjugate in infected plant tissues [16, 62]. Maize cell suspension cultures can modify ZEN after reduction to its phase I metabolites, α-ZEL and β-zearalenol (β-ZEL) and produce glucose conjugates of the respective compounds, especially Z14G. *Arabidopsis thaliana* can transform ZEN and its metabolites into a multitude of extractable conjugated compounds, including glucosides, malonylglucosides, dihexosides and pentosylhexosides [63].

To date, only a few studies have demonstrated the presence of these metabolites in crop plants. A survey of 10 wheat grain samples revealed the relative proportion of Z14G to ZEN to be on average about 27% [64]. In a survey of extractable conjugated *Fusarium* mycotoxins in cereal-based raw materials and finished products, none of the 84 cereal-based products analysed contained Z14G, α- or β-ZEL, α-zearalenol-14-β-D-glucopyranoside (α-ZELG) or β-zearalenol-14-β-D-glucopyranoside (β-ZELG) [48]. However, zearalenone-14-sulfate (Z14S) was found in different commodities (wheat flour, whole-meal wheat bread, maize meal, biscuits, wheat flakes, bran flakes, muesli, crackers, cereal snack bars and polenta), albeit in low concentrations, with the highest quantity being 6.1 μg/kg in bran flakes [48]. Thirty samples of a variety of food and feed matrices including maize, wheat, oats, cornflakes and bread were analysed for ZEN, α- and β-ZEL, Z14G, α-ZELG, β-ZELG, Z14S [47]. The incidence of ZEN in food and feed matrices was 80%. α-ZEL and β-ZEL, respectively, occurred in 53 and 63% of the samples. Z14G was detected in nine samples from trace levels up to 274 μg/kg. In one maize sample, the co-occurrence of Z14G (274 μg/kg), Z14S (51 μg/kg), β-ZELG (92 μg/kg) and the relatively low amount of ZEN (59 μg/kg), suggested that approximately 90% of the available ZEN was metabolised.

The occurrence of acyl conjugates of ZEN has been described for infected banana fruits [59] but the result appears to be an artefact [60].

### 3.3 Masked fumonisins

Several studies have reported the presence of bound fumonisins in food, which can be determined only after application of a hydrolysis step [65–69]. In particular, it has been observed that after performing alkaline hydrolysis of contaminated corn products (e.g. extruded products such as corn flakes) the amount of fumonisins released was often higher than the stoichiometrically expected value.

Several authors have reported the possibility of covalent bond formation between the tricarboxylic moiety and hydroxyl groups of carbohydrates or the amino groups of amino acids upon heating [70–75]. However, another masking phenomenon, based on a probable physical entrapment of the mycotoxins into the structure of macromolecular components (such as starch) [65, 66, 76], may have a strong influence on the accuracy of fumonisin measurement. Thus, other masking mechanisms such as complexation or physical entrapment should be taken into account when evaluating the occurrence of bound fumonisins. Dall'Asta and coworkers also reported the occurrence of bound fumonisins in raw maize and suggested that such non-covalent interactions were responsible for the phenomenon [69]. In order to further investigate the fumonisin-matrix interaction in raw maize, an in vitro digestion model was applied to raw maize and maize-based product to evaluate the possible release of bound fumonisins by enzyme-driven matrix disaggregation [77]. Upon digestion of the food matrix, a large increase of the total detectable fumonisins was observed in comparison with the analysis on the non-digested matrix. The release of parent forms of fumonisins in the case of raw maize was in agreement with an associative nature of the masking mechanism. These findings were in agreement with those already reported [78, 79].

The occurrence of bound fumonisins in commercial cornflakes was first reported in 2003 [65]. Samples of retail corn flakes were analysed for both free fumonisins and protein-bound fumonisins, which were extracted with SDS and measured as hydrolyzed fumonisin B_1_ (FB_1_) after alkaline hydrolysis. On average, a 2.6 times higher content of FB_1_ was found after hydrolysis. A similar approach was applied to 30 retail samples of heat-processed corn foods, revealing bound FB_1_ in all samples at significant levels [66]. The occurrence of bound fumonisins in 21 gluten-free products, at concentration levels comparable or higher than those found for the parent forms, has also been reported [68]. Ninety-seven maize samples collected in Italy were analysed for free and total fumonisins just after harvesting [77]. Free FBs were found in all samples at concentration levels ranging from 0.05 to 40 mg/kg, with a median value of 3.52 mg/kg after correction for moisture content. Total FBs obtained after alkaline hydrolysis were in the range 0.05 to 69 mg/kg, being significantly higher than free fumonisins in 82 of 97 samples.

Fumonisin fatty acid esters were identified in rice cultures inoculated with *F. verticillioides* – these substances are, however, most likely to have been fungal metabolites [80]. Nonetheless, fatty acids seem to play an important role in the formation of hidden fumonisins in planta, as described recently [81]. The chemical composition of several maize hybrids was analysed and compared to fumonisin contamination. A relationship between the amount of bound fumonisins and the ratio of oleic and linoleic acid in maize was observed.

### 3.4 Other masked mycotoxins

Plant metabolism of OTA was first studied using cell suspension cultures of wheat and maize incubated with ^14^C-OTA [82]. In addition to ochratoxin α, the main metabolites isolated were (4R)- and (4S)-4-hydroxy-ochratoxin A. In addition, β-glucosides of both isomers were found in large amounts. Ochratoxin α is commonly regarded as non-toxic, whereas hydroxy-ochratoxin A is an immunosuppressant as effective as OTA itself. The toxicity of the other derivatives is still unknown. The ability of crops to metabolise OTA was investigated using cell suspension cultures of several plants [83]. The isolated derivatives were the same for all the species tested and the conversion of OTA was nearly complete, although the quantitative distribution differed strongly depending on the plant. These transformations were also seen in germinating cereals and vegetables after addition of OTA [84]. No studies have been published to evaluate whether or not these derivatives also occur in naturally contaminated food.

One study reported the possible occurrence of bound patulin in apple juice [85]. In particular, a decrease in patulin recovery was observed during storage when cloudy apple juice was spiked with this mycotoxin. The decrease was significantly more pronounced for lower spiking levels. The authors hypothesised an interaction between the solid part of the juice and patulin. This was also in agreement with the previously reported observation that patulin contamination of cloudy apple juice can be reduced upon clarification and that the solid residue becomes enriched with patulin [86]. Since patulin is able to undergo an electrophilic attack on molecules containing a nucleophilic group, in particular with proteins or small peptides containing cysteine, lysine or histidine residues [87], a binding between this compound and the solid part of cloudy apple juice may be supposed. This covalent binding cannot be cleaved during extraction, leading to an underestimation of the overall patulin content. Although toxicity data are not available for bound patulin, the conjugation of the electrophilic group may lead to a loss of toxicity.

Destruxins are cyclic hexadepsipeptides produced by certain species of the fungal genera *Metarrhizium*, *Alternaria* and *Trichothecium,* which are toxic to a wide range of invertebrates and plants [88]. Although less effort has been invested in studies of vertebrate toxicity of destruxins, their cytotoxicity has been demonstrated in murine leukaemia cells, spleen lymphocytes and other targets. Destruxins might therefore be regarded as mycotoxins. Cruciferous crops hydroxylate destruxins and conjugate the hydroxylated derivatives to glucose [88].

Fusaric acid is one of the oldest known mycotoxins. It exerts neurotoxic effects in mammals [89] and possesses pharmacological activities [90]. Transformation of fusaric acid by many plant species to its N-methylamide derivative was documented extensively half a century ago (reviewed in [91]).

## 4 Analytical aspects

A large variety of analytical methods is used for mycotoxin determination in foodstuffs, including chromatographic methods such as TLC, GC or LC and immunochemical methods such as ELISA. Immunochemical methods can respond to more than one compound (e.g. a mycotoxin and its derivative) leading to a single result. This depends on the cross-reactivity of the antibody to these derivatives [92]. Chromatographic methods on the other hand resolve each compound (in an ideal case) as single parameter. Biological methods that only intend to measure a common effect related to a class of substances have been developed in other fields [93, 94].

All analytical methods for parent mycotoxins are potentially also suitable for their conjugated forms. However, it should be emphasised here that only soluble analytes are directly accessible for analysis. Bound (immobilised or insoluble) forms cannot be detected without sample treatment that converts them into soluble forms, while soluble conjugated forms can be determined after extraction with typical solvents used in mycotoxin determination. Several techniques are available for the conversion of mycotoxin metabolites into their parent forms. After hydrolysis, any method capable of determining parent mycotoxins can be expanded to include mycotoxin metabolites, even if analytical standards for the conjugated varieties are not available. A drawback of indirect methods is that the efficiency of hydrolysis can only be estimated by monitoring residual signals of structurally identified mycotoxin conjugates after hydrolysis. Furthermore, these methods cannot discriminate between different conjugates of the same parent toxin.

### 4.1 Extraction and clean-up

Most analytical processes require the isolation of the target analyte from the matrix for further determination [95] with a few exceptions such as direct spectroscopy [e.g. [96]. For the most commonly used methods such as chromatography, fluorimetry and ELISA, extraction is unavoidable. Extraction should be quantitative, preferably specific to the (group of) target analytes and compatible with the analytical method used.

Transformation of masked mycotoxins into parent molecules involves hydrolysis [52, 97], the type of which has to be selected with care. Some masked mycotoxins can be subjected to alkaline hydrolysis [67], while for others such as DON this is not the case [98]. Transformation of food contaminants has also been applied in other fields for risk analysis where a substantial number of related substances is too challenging to be analyzed individually [99]. For monitoring purposes, it will be advantageous to determine the total amount of a mycotoxin (parent and conjugate) either by a common response (e.g. by ELISA) [92] or after transformation to a single analyte [52, 67].

#### 4.1.1 Extraction conditions for direct methods

Mycotoxins and their metabolites are soluble in mixtures of polar organic solvents (ACN or methanol (MeOH)) with water. After shaking for 30 to 90 min, the extracts are filtered and analysed with or without prior SPE clean-up. ACN:water 84:16 (v/v) was used for the extraction of Z14G [64] and D3G [46] from cereals. Sulyok and coworkers added acetic acid (HOAc) in the proportions ACN:water:HOAc 79:20:1 (v/v/v), and reported that these conditions were suitable for the determination of many mycotoxins, including D3G, in wheat and maize 100. De Boevre and coworkers used of the same solvent in combination with a hexane defatting step, for simultaneous extraction of DON, ZEN, T-2 and ten (masked) metabolites thereof 47. It was reported that neutral and acidic extraction gave similar results for D3G, 3ADON, Z14G, α-ZELG and β-ZELG [101]. Using a higher proportion of water reduced the recovery of non-polar toxins. Acidic conditions slightly improved the recovery of DON and significantly improved the yield of ZEN. Centrifugation was compared with shaking to extract mycotoxins from wheat using MeOH:dichloromethane (50:50, v/v) or ACN:water (84:16, v/v) being used as extraction solvents [42]. Centrifugation improved extraction efficiency and MeOH:dichloromethane provided higher recoveries than ACN:water. Further advantages of the former solvent were minimisation of matrix effects and extraction of both polar and less polar mycotoxins, however the use of chlorinated solvents is discouraged today.

### 4.1.2 Clean-up for direct methods

Commercially available clean-up columns for mycotoxins include SPE, often with multifunctional modes, and immunoaffinity columns (IACs). However as extractable conjugated mycotoxins are mostly more polar than their parents, SPE clean-up techniques might not be suitable. As more sensitive LC-MS/MS equipment has become available, it has become possible to replace clean-up by diluting the sample extracts [8]. Typically, extracts obtained from the ACN:water:HOAc solvent system were suitable for LC-MS/MS analysis without further clean-up [100].

De Boevre tested four SPE cartridges and showed that no acceptable recoveries were obtained for masked mycotoxins [47]. Previously, eight commercially available clean-up cartridges and primary/secondary amines (PSA) were compared [101]. The IACs tested did not retain glucosylated compounds or Z14S. The C18 SPE phase resulted in better recoveries of 3ADON, Z14G, α-ZELG and β-ZELG, but for Z14S the recovery was reduced to 21%, less than the low recovery (43%) for the untreated extract. D3G and DON were not recovered by C18 SPE under the used conditions. PSA brought no advantage. Sasanya and coworkers used Phenomenex Strata-X® clean-up using increasing concentrations of MeOH for elution, followed by MeOH:ACN:water:HOAc [42]. All fractions contained D3G, resulting in a sample clean-up that was an unsuitable for D3G. Florisil column clean-up was also applied to the measurement of Z14G in wheat extracts [64]. After extraction with MeOH:tert-butyl-methyl ether, the sample was placed on the column, which was then was washed with hexane and Z14G eluted with MeOH:ethyl acetate. Table 1 gives an overview of extraction and clean-up methods used for masked mycotoxin analysis.

**Table 1:** Variety of extraction and clean-up for direct methods

Matrix	Analyte	Solvent	Time (min)	Clean-up	Reference
Wheat/maize	D3G	ACN:water, 84:16	90	Mycosep® 230	[37]
Wheat	D3G	MeOH:dichlormethane, 50:50	30	Strata-X®	[42]
Malts	D3G	ACN:water, 84:16	60	Mycosep® 226	[45]
Malts	D3G	ACN:water, 84:16	60	None	[46]
Maize	D3G	ACN:water:HOAc, various	90	None	[101]
Wheat	D3G	ACN:water, 84:16	60	Various ELISA	[126]
Wheat	Z14G	ACN:water, 84:16	1	Florisil	[64]
Maize	Z14S	ACN:water:HOAc, various	90	None	[101]
Maize/wheat/oats/	D3G, ZELGs,	ACN:water:HOAc, 79:20:1	60	Hexane defatting	[47]
bread/cornflakes	Z14G, Z14S, etc.				

#### 4.1.3 Hydrolysis in indirect methods

Indirect methods have the advantage that chemical standards of the conjugated forms are not required and are likely to account for various uncharacterised conjugated forms that might be missed by targeted analysis. Conversely, without authentic standards of the conjugates the efficiency of the hydrolysis process cannot be determined. Hydrolysis of the conjugated mycotoxins can be achieved by enzymatic, acidic or basic treatments (summarised in Table 2). Unfortunately no current single hydrolysis method is applicable to all masked mycotoxins.

**Table 2:** Summary of indirect methods

Matrix	Analyte	Pre- hydrolysis extraction	Hydrolysis	Temp (°C)	Time (min)	Post hydrolysis extraction	Clean-up	Reference
Barley	DON	ACN:water	TFA	133	70	–	C18 SPE	[52]
Corn	DON	water	TFMSA	22	20	–	–	[53]
Wheat	DON	water	TFMSA	40	40	–	–	[53]
Wheat	DON	ACN:water	TCA	140	40	–	MycoSep® SPE	[104]
Corn	FBs	SDS	2N KOH	60	60	EtOAc	None	[65]
Corn cereal	FBs	SDS	2M KOH	60	60	MeOH:EDTA	HLB®	[66]
Tortilla/chips	FBs	SDS	2M KOH	60	60	MeOH:ACN:water	HLB®	[66]
Corn	FBs	MeOH:water	2M KOH	RT	10	ACN	–	[69]

SDS solution is a useful solvent for dissolving proteins and has been used to extract bound fumonisins [70]. Bound FB_1_ is cleaved from the matrix and determined as hydrolyzed FB_1_. This method was improved by introducing a water wash step to remove residual parent fumonisin [65]. Hydrolyzed FB_1_ was isolated using Oasis HLB® columns, as it had been shown previously that C18 cartridges were ineffective [102]. The water wash was supplemented with a solvent and protein-bound and total fumonisins determined separately by extracting protein-bound FB_1_ with SDS [66]. The presence of SDS shifts the chromatographic retention time, making LC-MS determination unreliable [65], but it can be removed by complexation with methylene blue prior to clean-up using an Oasis HLB® column [66].

ß-glucosidase has been used to cleave Z14G, releasing ZEN [7]. Unfortunately a large excess of enzyme is required and reaction times are lengthy (18 h). The ZEN released from Z14G is measured together with the parent ZEN, typically by LC with fluorescence detection. Glucosidases are in general not effective against glucosides of all mycotoxins, including D3G [36] and so their application is limited. Several other enzymes or enzyme mixtures have been used. Enzyme treatments with amylolytic (α-amylase, amyloglucosidase), proteolytic (papain) or cell wall degrading (cellulose, xylanase) enzymes had a significant effect on the quantity of DON released from its bound forms in barley [103]. Amylases increased the level of DON measured by 28% and papain increased it by 19%. Cellulase had only a small effect.

Fumonisins will lose their conjugated side chains upon treatment with strong bases. As sugar, starch, peptide or protein conjugates are also attached to the side chains, fumonisins can be liberated by this treatment and measured [102]. For example, a 2.6-fold increase of ‘total’ fumonisins in cornflakes after treatment with 1% SDS solution and hydrolysis with 2M KOH was found [65]. Several authors have also described the alkaline hydrolysis of fumonisin conjugates [66, 69].

Acid hydrolysis procedures based on hot trichloroacetic acid (TCA) were applied to samples containing 3ADON and 15ADON [104]. The method was further optimised substituting TCA with TFA [52]. The procedure did not hydrolyse all of the ADON known to be present. Also, it is unclear whether D3G is fully converted to DON. Recently, the optimal conditions in which conjugated DON in corn and wheat could be hydrolyzed by TFMSA were determined [53].

### 4.2 Chromatographic methods

TLC methods are fast, easy, cheap but also quite insensitive. TLC is sometimes used for the analysis of cultures of potentially toxigenic fungi isolated from food. TLC is still used for mycotoxin analysis in developing countries (reviewed in [105]). As masked mycotoxins usually occur in lower concentrations than their parent forms, TLC does not appear suitable for their determination.

GC methods exist for the quantification of trichothecenes (reviewed in [106]), ZEN (reviewed in [107]), OTA (reviewed in [108]) and fumonisins [109]. Derivatisation is needed to render the mycotoxins volatile. While conceivable, no GC methods for extractable conjugated mycotoxins are known today. As extractable conjugated (especially glucosylated) mycotoxins are even more polar than their parent forms, and so excessive derivatisation would be needed.

All current chromatographic methods for the determination of masked mycotoxins are based on LC. Fluorescence detection is available for compounds with a natural fluorescence, such as derivatives of ZEN (e.g. Z14G, [7]) or OTA. However, LC-MS is the method of choice (e.g. [37, 63, 101]). The majority of LC-MS methods relies on simple dilute and shoot strategies and reversed-phase chromatography is used for most masked mycotoxins. The Synergi® reversed-phase C18 stationary phase gave a relatively even distribution of extractable conjugated *Fusarium* toxins [101]. HILIC methods can also conceivably separate very polar compounds. De Boevre tested three columns with HILIC properties (XBridge® HILIC, Discovery® HS F5 and TSKgel® Amide-80), however, no satisfactory retention of polar compounds such as D3G was obtained [47]. LC stationary phases applied so far for masked mycotoxin analysis are shown in Table 3.

**Table 3:** Overview on LC columns, solvents and MS mode

Analyte	Column	Solvent	MSa)	Mode	Reference
D3G	Aquasil® RP18 100 × 4.6 × 3	MeOH:water, 85:15	QTrap® MS/MS	−APCI	[37]
D3G	Synergy® fusion 150 × 4.6 × 4	MeOH:water, 70:30	QP8000® MS/MS	+Full scan	[42]
D3G	Gemini® C18 100 × 4.6 × 5	MeOH:HOAc:AA	QTrap® MS/MS	−ESI	[43]
D3G	Synergy® hydro RP 100 × 3 × 4	MeOH:water:AA	LCQ® MS/MS	±APCI	[45]
D3G	Synergy® hydro RP 100 × 3 × 4	MeOH:water:AA	LCQ® MS/MS	±APCI	[46]
D3G	Synergy® hydro RP 150 × 3 × 4	MeOH:water:AA	LCQ®	−APCI	[126]
ZEN conjugates	Aquasil® RP18 100 × 4.6 × 3	MeOH:AA gradient	QTrap® MS/MS	−ESI	[63]
Z14G	Nucleosil® C18 120 × 125 × 2	ACN:formic acid	VG® Quadrupole	+ESI	[64]
Multi	Synergy® polar RP18 150 × 4.6 × 5	ACN:HOAc:AA	QTrap® MS/MS	−ESI	[101]
Multi	Gemini® C18 150 × 4.6 × 5	MeOH:HOAc:AA	QTrap® MS/MS	±ESI	[100]
Multi	Zorbax® XDB C18 100×4.6×3.5	MeOH:water:AA	Quattro Premier XE® MS/MS	+ESI	[47]

AA, ammonium acetate.

a) Manufacturer's abbreviated name for the system.

ESI is often employed to ionise polar mycotoxin metabolites. This is crucial for charged metabolites (e.g. Z14S), as atmospheric pressure chemical ionisation (APCI) cannot transfer charged ions into the gas phase. APCI has been used for the ionisation of D3G [37, 45, 46, 51]. Collision-induced dissociation of extractable conjugated mycotoxins in MS/MS often yields the (unconjugated) parent toxin ion or fragments thereof. Method performance is comparable to methods for parent toxins, but matrix effects are sometimes more severe [101]. TOF-MS has a high resolving power and can give methods that are sensitive in full scan modes. They can target analytes but also provide empirical formulae of unknown compounds. Tandem MS can help identifying mycotoxin conjugates through precursor ion and neutral loss scans.

While the majority of the methods described in this review are based on MS, other detection methods might be used. The determination of DON based on HPLC with post column derivatisation [110] appears such an option. Since this reaction works also for other type B trichothecenes such as nivalenol as well as acetylated derivatives, it may also be suitable for other extractable conjugated DON derivatives. Such methods would allow, upon availability of reference substances, the determination of extractable conjugated DON derivatives without the need of MS.

Finally, chromatographical methods for the determination of masked mycotoxins, including strategies to detect currently unknown forms have recently been reviewed [111].

### 4.3 Immunochemical methods

ELISA methods continue to be widely used for fast screening of commodities and foods for mycotoxins due to their relatively low cost and easy application [112]. Commercial ELISA kits, lateral flow test strips (LFDs) and IAC for sample clean-up are widespread, making use of specific and well-characterised polyclonal or monoclonal antibodies [113]. To date, no antibodies have been specifically targeted and selected against masked mycotoxins. Antibodies developed against the parent mycotoxin can potentially cross-react with masked forms if the epitope is not sterically hindered by the metabolisation [8, 114]. Only a few studies on the evaluation of ELISA for the recognition of masked mycotoxins have been performed. Indeed, data exist on the cross-reactivity of DON antibodies for 3ADON, 15ADON, nivalenol and other structurally related trichothecenes [115, 116]; on the cross-reactivity of ZEN antibodies for α-ZEL, β-ZEL, zearalanone, α-zearalanol and β-zearalanol [117]; on the cross-reactivity of OTA antibodies for ochratoxin B [118]; on the cross-reactivity of FB_1_ antibodies for FB_2_ and FB_3_ [119]; on the cross-reactivity of T-2 antibodies for HT-2 and other related trichothecenes [120] and on the cross-reactivity of AFB_1_ antibodies for AFB_2_, AFG_1_, AFG_2_ and AFM_1_ [121, 122] while masked forms have mostly been neglected.

Because of the commercial availability of D3G reference standards, most attention has been paid to this masked mycotoxin. A surface plasmon resonance immunoassay was developed using a monoclonal antibody that showed 60% cross-reactivity with D3G [123]. Cross-reactivity studies with D3G were performed for different commercially available DON enzyme immunoassay kits as shown in Table 4. The measured cross-reactivity versus the one declared by the manufacturers was compared [124]. Most of them did not report cross-reactivity values for D3G, while for Veratox® (Neogen Corp., Lansing, MI, USA) 0% was declared and 157% measured. Also for other compounds, such as 3ADON, large differences were observed between declared and measured cross-reactivity. It should be noticed that the experiments in this study were performed in pure water. Differences observed can be the result of the different methods used for the cross-reactivity assessment. Also, it is suggested that cross-reactivity with spiked matrix extracts are evaluated while checking the matrix effect on cross-reactivity results. Concerning fumonisins, different FB_1_ derivatives have been checked for cross-reactivity with a fumonisin antibody by using the Ridascreen® fumonisin assay (R-Biopharm) [67]. Knowledge on cross-reactivity of ELISA kits is crucial for correct data interpretation. The establishment of clear guidelines by international standardisation bodies on how to perform cross-reactivity studies for commercial ELISA kits is highly recommended. Due to unknown or poorly characterised cross-reactivity with masked mycotoxins, ELISA assays can result in over-estimation of a specific mycotoxin contamination. However, they may give an idea about the total amount of the target compound together with other co-existing analogues that can indeed be useful for overall risk assessments. In general, ELISA tests should only be used as screening techniques and positive findings should be confirmed by a more specific analytical method such as LC-MS/MS.

**Table 4:** Cross-reactivities of commercial enzyme immunoassay kits and IACs for masked mycotoxins

Commercial kit	Supplier	Studied cross-reacting masked mycotoxin	Cross- reactivity (%)	Reference
**ELISA**				
Ridascreen® DON	R-Biopharm	D3G	82–98	[92]
Agraquant®	RomerLabs	D3G	52	[124]
DON EIA®	Europroxima	D3G	115	[124]
Veratox®	Neogen Corp.	D3G	157	[124]
Rosa LF-DONQ® (LFD)	Charm	D3G	8	[124]
MycontrolDON® (Fluorescence	Aokin	D3G	22	[124]
polarisation IA)				
Ridascreen® fumonisin	R-Biopharm	Hydrolyzed FB_1_	0	[67]
Ridascreen® fumonisin	R-Biopharm	Protein extract containing bound fumonisins	Positive for all analytes, but not quantified	[67]
**IAC**				
DON-Prep®	R-Biopharm	D3G, Z14G, α-ZELG, β-ZELG, Z14S	0	[101]
DON test®	Vicam	D3G, Z14G, α-ZELG, β-ZELG, Z14S	0	[101]
Easi-extract® ZEN	R-Biopharm	D3G, Z14G, α-ZELG, β-ZELG, Z14S	0	[101]
Zearatest®	Vicam	D3G, Z14G, α-ZELG, β-ZELG, Z14S	0	[101]
Zearastar®	RomerLabs	D3G, Z14G, α-ZELG, β-ZELG, Z14S	0	[101]
DON-Prep®	R-Biopharm	D3G, Z14G	58, 0	[125]a)
NeoColumn® DON	Neogen Corp.	D3G, Z14G	48, 0	[125]a)
ImmunoClean® C DON	Aokin	D3G, Z14G	0	[125]a)
Easi-extract® ZEN	R-Biopharm	D3G, Z14G	0	[125]a)
NeoColumn® ZEN	Neogen Corp.	D3G, Z14G	0	[125]a)
ImmunoClean® C ZEN	Aokin	D3G, Z14G	0	[125]a)
DZT® MS-prep	R-Biopharm	D3G, Z14G	41, 0	[125]a)

a) Further tested mycotoxins were: 3ADON, 15ADON, DON-3-glucuronide, ZEN-glucuronide, deepoxy-DON, α-ZEL, β-ZEL and nivalenol.

Also for IACs, cross-reactivity with masked forms depends on the immobilised antibody. Therefore, existing IACs should be evaluated for binding mycotoxin conjugates [8]. In two studies [101, 125], several commercially available DON and ZEN IACs were tested for cross-reactivity to DON and ZEN derivatives (Table 4).

### 4.4 Comparison of methods

The three major approaches to the determination of masked mycotoxins are indirect determination, direct analysis by chromatography and direct analysis by ELISA. The direct and indirect methods have different applications. Indirect methods provide a relatively rapid measure of the total mycotoxin content but cannot differentiate between free and masked mycotoxins, and the hydrolysis conditions used (enzyme or acid) might be either inadequate or destructive. The methods have an advantage in that reference standards of the masked mycotoxins are not essential. Direct methods are based on the two very different principles of chromatographic separation and immunoassays.

Data obtained by four commercially available DON dedicated ELISA kits and LC-MS/MS were critically assessed for use in malt and beer [126]. Cross-reactivities of DON conjugates were evaluated in aqueous solution and in spiked beer. Besides high cross-reactivity with 3ADON for all kits, cross-reactivity with D3G was shown ranging from 32 to 78% in water and from 51 to 104% in beer. The authors analysed 20 beer samples obtained at the European market and showed that apparent DON levels obtained by all immunoassays were significantly higher than LC-MS/MS values. D3G and ADONs contributed to DON overestimation by ELISA. However, the poor correlation between the gained values also indicated the presence of other, unknown cross-reacting compounds. For instance, the likely presence of DON di- and tri-glycosides in beer could contribute to the total overestimation. A survey involving in total 176 beers has documented almost ubiquitous occurrence of D3G [46]. Comparison between LC-MS/MS and ELISA DON kits showed the latter provided apparently higher levels of DON, the most distinct difference being observed for malts processed at higher temperatures. That phenomenon might be explained either by matrix compounds, or by unknown DON related compounds formed at elevated temperatures. Discrepancies between ELISA and LC-MS/MS results for DON analysis in wheat were also reported in other studies [45, 127].

The relative performance of individual LC-MS/MS methods can be compared only with difficulty as they rely on different extraction and clean-up procedures. The typical performance of LC-MS/MS methods in terms of LOD, LOQ, recovery and repeatability is shown in Table 5. In general, recoveries are mostly acceptable and LOQs are often in the low microgram per kilogram range.

**Table 5:** Comparison of full method performance for direct LC-MS/MS methods

Toxin	Other analytes	Matrix	LOD (μg/kg)	LOQ (μg/kg)	Recovery (%)	Repeatability (RSD_r_,%)	Reference
D3G	DON	Wheat	1	0.5	70	8	[42]
D3G	DON	Wheat	4	10	71	10	[43]
D3G	DON	Maize	4	10	130	10	[43]
D3G	7	Brewery malts	0.5	5	34–50	<10	[45]
D3G	7	Malts	N/A	1	85	5	[46]
D3G	7	Beers	1	2.5	81	6	[46]
D3G	38	Maize	4		66	5–17	[100]
D3G	9	Cereal foods	50–100	100–250	50–90	6–11	[101]
D3G	12	Maize	8	16	85	18	
		Wheat	8	16	90	8	
		Oats	7	14	93	12	[47]
		Cornflakes	12	24	103	5	
		Bread	13	26	97	8	
Z14G	ZEN	Maize	-	10	67–69	-	[64]
Z14G	38	Maize	2		100	5–22	[100]
Z14S	38	Maize	0.1		79	3–8	[100]
Z14G	9	Cereal foods	4–10	10	100–108	4–15	[101]
Z14S	9	Cereal foods	0.5–1	1–10	67–103	5–9	[101]
ZELGs	9	Cereal foods	4–10	10–25	91–111	7–10	[101]
Z14G	12	Maize	7	14	85	21	
		Wheat	8	16	89	24	
		Oats	9	18	88	25	[47]
		Cornflakes	10	20	83	26	
		Bread	10	20	83	24	

### 4.5 Reference materials

Currently, research about masked mycotoxins is hampered by the non-availability of analytical standards or calibrants. Only one compound – D3G – is commercially available at the time of this report. Standards are essential for direct methods and for the quantification of masked mycotoxins, but not necessarily for screening or indirect methods. Often, masked mycotoxins are either isolated (e.g. D3G [37]) from inoculated plants or chemically synthesised (e.g. Z14G [128]) from the parent toxin by research groups on their own. Also biosynthetic methods for glucosylation are known [16, 129]. For example, an engineered yeast strain, expressing a UGT, was fed with ZEN to yield its glucoside, which was then purified from the supernatant. While most substances are stable in solid form, the stability of extractable conjugated mycotoxins in solution is critical. For instance, 3ADON and Z14S are prone to decay to their parent toxins in aqueous or methanolic solutions. The use of aprotic solvents (e.g. ACN) improves the stability of standards.

## 5 Toxicological considerations

As masked toxins present an emerging issue it is not a surprise that for most toxicological study types – such as genotoxicity, short-term and long-term toxicity including carcinogenicity, reproduction and developmental studies – no data have been made available. As conjugation is known to be a detoxification process, it seems likely that conjugated mycotoxins exhibit a lower acute toxicity compared to their parent compounds. This is demonstrated with data for D3G, which showed a dramatically reduced ability to inhibit protein synthesis of wheat ribosomes in vitro compared to DON [38]. Also, Z14G yields far lower estrogenic activity compared to ZEN [16].

No specific bioavailability studies have been performed with masked mycotoxins so far. Based on the present knowledge about mycotoxins and absorption of xenobiotics it can be assumed, that conjugation might change their bioavailability. This matter is especially intriguing in the case of fumonisins, which are very poorly absorbed in their parent form.

Just a few specific metabolism studies with masked mycotoxins are available. Gareis et al. demonstrated that Z14G is decomposed during digestion and the aglucone, ZEN, is released in pigs [7]. In pigs and probably in humans, ZEN is rapidly absorbed after oral administration and can be metabolised in intestinal cells (reviewed in [27]). There, ZEN is degraded into α-ZEL and β-ZEL, which are subsequently conjugated with glucuronic acid and excreted in the urine [27, 130]. Judging from this data, it appears likely that the toxic (estrogenic) effects of Z14G equal that of ZEN in mammals. As the bioavailability of ZEN is already very high, a possible increased bioavailability of Z14G should not be relevant. Conversion of Z14G to ZEN seems to be rather exhaustive. In this case, a (molar) sum parameter for ZEN and Z14G might very well work to describe to potential health threats of contaminated food.

Recently, a study described the hydrolytic fate of D3G during digestion in vitro [131]. D3G was found resistant to hydrochloric acid, suggesting that it will not be hydrolyzed in the stomach of mammals. While human cytosolic β-glucosidase also had no hydrolytic effect, several lactic acid bacteria, isolated from guts, such as *Enterococcus durans*, *E. mundtii* or *Lactobacillus plantarum* liberated DON from its glucoside. While the potential cleavage of D3G during digestion has been shown, in vivo studies are necessary to describe DON liberation from D3G in a more quantitative manner. Preliminary results suggest that D3G is partly cleaved to DON which was found along with its glucuronide in rat urine [132]. Reviews on the metabolism and toxicological mechanisms of trichothecenes in human and animals are available [26, 133, 134]. DON bioavailability appears to be low in sheep and cows, but relatively high in pigs [135]. It will be interesting to see if D3G shows an increased uptake compared to DON and if its cleavage is exhaustive or only partial, as D3G seems to be the more stable glucoside compared to Z14G.

No specific toxicological studies on bound fumonisins have been performed so far. There is little evidence that fumonisins are metabolised in animals, even though they are clearly excreted in bile. Fumonisins are excreted primarily in faeces, either unchanged or with loss of one or both tricarboxylic acid side chains [136]. The poor absorption of FB_1_ might be increased for bound forms.

Also for OTA, no specific toxicological studies on its plant metabolites were conducted. The Joint FAO/WHO Expert Committee on Food Additives (JECFA) evaluated the toxicity of OTA in 2001 137. The toxicokinetic and toxicodynamics have been reviewed [138]. The bioavailability of OTA in two mammalian species was quite high at 44–99% [139]. The occurrence of OTA metabolites in food is unverified. If such substances occur and are converted to OTA during digestion, an additive scenario of the toxic effects are likely as the absorption of OTA already is high.

It can be concluded that at present there are no toxicokinetic and toxicodynamic studies available, which would enable hazard or risk assessment of masked mycotoxins in comparison with those of the parent mycotoxins. Despite a low general toxicity of the few investigated substances, possibly increased bioacessibility and partial reactivation of masked mycotoxins during digestion in mammals remain a health threat.

## 6 Impact on stakeholders

### 6.1 Food processes with potential influence on mycotoxin conjugates

Many agricultural products that are subject to mycotoxin contamination are consumed by animals, but are not consumed as such by humans. Often they are processed using fermentation, chemical hydrolysis or germination (e.g. barley). The use of living cells, and enzymes as well as alkaline or acidic hydrolytic conditions during processing can lead to the liberation or generation of masked mycotoxins.

Two fermentation processes are relevant in food industry; production of microbial cells and transformation of the source material (e.g. production of beer, wine or bread) [140]. The production of microbial cells for food production is limited to Quorn, a myco-protein made from *F. venenatum* strain PTA-2684 grown on a glucose source [141]. During product transformation processes a variety of enzymes are involved, principally amylases (e.g. baking, brewing, coffee fermentation, corn syrup production), proteases (e.g. baking, brewing), pectinases (e.g. bread, coffee), β-glucanases (brewing) and amyloglucosidase (corn syrups). These enzymes are expressed by living cells fermenting the product. In case of beer production, two different steps play a role; germination and yeast fermentation. In this context, it has been shown that levels of D3G readily exceed DON levels in beer [46]. As the levels of D3G are already higher than those of DON in malt, germination has an important role. The production of traditional soy sauce is also a two-step fermentation process, where *Aspergillus* fermentation is followed by yeast fermentation. Soy sauce is frequently not only produced from soy alone but also incorporating wheat. Industrial cost efficient production of such soy sauces also makes use of acid hydrolysed ingredients [142]. No data on mycotoxin conjugates is available on soy products.

The production of bread requires either fermentation of wheat flour with yeast or rye flour with leavening agents (leaven). Two recent studies reported the fate of D3G within milling and baking [143, 144]. About 20–60% of both D3G and DON could be removed during the production of white flours from grains, according to the study. The addition of enzymes mixtures as bakery improvers gave a rise of up to 145% of the level of D3G in fermented dough. Baking slightly decreased the levels of D3G and DON and thermal degradation products of DON were found – mostly located in the bread crusts. A follow-up experiment showed that DON levels in flour were significantly higher after treatment with protease (16%) and xylanase (39%) [144]. Also of interest is the production of rye bread with leaven, as this is a process that requires a more extensive enzymatic activity, typically of up to several days [145]. To date, no information on the effect of rye fermentation on mycotoxins and their conjugates has been described in the literature. Similarly, a Canadian study reported already in the mid 1980s that the DON content of doughnuts fermented with yeast was higher than in the flour used, which was likely to be due to the co-occurrence of D3G, from which DON was partially liberated during the processing [146].

Isolated enzymes such as bacterial α-amylase (as endoenzyme), fungal glycosidase (as exoenzyme) and bacterial pullulanase are used for the production of glucose syrups and high dextrose equivalent syrups for the beverage industry. These are obtained mainly from maize and include high fructose corn syrup [147]. These syrups can be processed further enzymatically and lead to a variety of products used in confectionery, jams, jellies, etc. [148, 149] as well as Quorn. Again, no information is available on masked mycotoxins in these products. Cocoa and coffee beans are subject to fermentation and the influence on mycotoxin production and fate during fermentation processes has been the focus of previous work [150–152].

Another important aspect is the fact that hydrolytic enzymes as well as micro-organisms are commonly used as animal feed additives. These are added, e.g. in order to liquefy the intestinal content or to increase the caloric yield of the feed [153]. To what degree masked mycotoxins are released by these additives has not yet been described. Other technological processes such as nixtamalisation of maize involve alkaline conditions for the improvement of its nutritional value (release of niacin from a bound to free form, available for absorption). It also reduces the amount of fumonisins due to hydrolysis, but the effects on other mycotoxins and possible conjugates have not been described. Modern nixtamalisation is a combination of alkaline treatment and enzyme treatment with proteases. Chemical hydrolysis will, by its nature, only be able to release masked mycotoxins, or may even lead to degradation of the mycotoxin. Alkaline degradation of DON and fumonisins is known.

The use of isolated hydrolytic enzymes might liberate mycotoxins from their masked forms. Also, isolated enzymes can be accompanied by other enzymes, therefore conclusions on the enzymatic capability of an isolated enzyme must be made carefully. Processes involving living cells are even more complex and involve the major hydrolytic enzymes in addition to enzymes needed for cell metabolism.

### 6.2 The analytical community

Since masked mycotoxins are not targeted by current methodologies for regulated mycotoxins, additional effort is needed to expand the methods and their use. Methods currently used for masked mycotoxins are all based on LC-MS (see above). The availability of methods suitable for control laboratories and the availability of suitable reference substances will be extremely important in the future. Therefore research analysts need to develop such methods including their performance criteria in order to allow the generation of consistent exposure data. The methods have to provide sufficiently low LODs and LOQs to avoid scenarios where misleading exposure data might be generated on account of too low sensitivity of the method.

### 6.3 Legislation

In 2010, JECFA considered D3G, 3ADON and 15ADON as additional contributing factor for dietary exposure to DON [154, 155]. JECFA experts identified a lack of toxicological data for D3G and recommended that due to the presence of D3G in food studies on absorption, distribution, metabolism and excretion are needed. Further occurrence data and effects of processing on the levels of D3G as well as ADONs are needed. As a result the Codex Committee on Contaminants in Food agreed to further develop the proposed draft maximum limits under discussion with regard to DON, allowing a further consideration by the next session of the Committee [156]. In addition, it encouraged members as well as industry to further monitor for the occurrence of DON and its derivatives.

In the EU, recent requests for evaluation of mycotoxins have been issued by the European Commission to the European Food Safety Authority (EFSA) for a range of mycotoxins, including ZEN (in breakfast cereals) and Alternaria toxins [157]. There is however at the moment no explicit plan for an evaluation for masked mycotoxins.

Taking into account that masked mycotoxins can be released during processing, a food commodity that has been correctly judged as compliant concerning the presence of the free mycotoxin could be judged as non-compliant at a later stage of production, due to release of the toxin. All scenarios, in which masked mycotoxins can be released during processing, have the potential to cause trade disputes. Therefore, possible trade disputes have to be addressed as well potential health concerns. In the European Union, this aspect is covered by Regulation (EC) No 178/2002 – the ‘general food law’ – which applies to all stages of the production, processing and distribution of food and also of feed [158]. The regulation also emphasises that next to a high level of protection of human life and health, the free movement of safe and wholesome food is an essential aspect of the internal market and contributes significantly to the health and well-being of citizens. Legislators need not only to consider this issue upon identification of a public health concern, but also the potential disturbances in trade. Both aspects will however require more scientific data prior to any conclusion being made.

As the study of masked mycotoxins is a rather young scientific topic, any maximum limits that might be drafted in the future should respect that masked mycotoxin study is likely to remain a dynamic scientific field and that legislation should take this into account in the same manner as the European Union has taken scientific progress in other areas into account, by regulating method performance criteria rather than community methods for mycotoxins [159]. A possible approach can be the definition of a parameter for the sum of all relevant forms of a mycotoxin, including its relevant derivatives.

## 7 Conclusion

Only few data has been collected so far concerning the occurrence of masked mycotoxins in food and/or feed. There is also a lack of information about their stability, transformation and release along the manufacturing chain. Specifically, the occurrence of masked forms of OTA, nivalenol, fusarenon-X, patulin, fusaric acid and destruxins has been described only in pioneer studies. Although the presence of masked forms of ZEN and fumonisins has been reported in cereal-based products, more occurrence data should be obtained, preferably through the implementation of market surveys. As far as D3G is concerned, its presence has been extensively investigated in cereals and cereal-based food, with reported D3G levels up to 70% of those of DON. Moreover, it has been shown that D3G in beer may even exceed DON levels. Although several studies indicated that D3G might be released from other conjugated forms during food processing, the fate of trichothecene conjugates along the food production chain should be further investigated.

The resistance of wheat towards *Fusarium* infection is partly linked to its ability to metabolise the plant pathogenic DON to D3G. One can assume that higher D3G/DON ratios will be accompanied by lower levels of total DON + D3G due to a better *Fusarium* resistance of crop plants. Therefore, a high ratio of D3G/DON might be a good bargain, if the total content of all DON metabolites is lower. This effect can however only be judged en gros for a large population of samples.

Analytical methods for the determination of masked mycotoxins can be based on direct procedures or hydrolysis to the parent forms. Hydrolysis can be achieved under alkaline conditions, or by using acids such as TCA or TFA, or with enzymes. The extractable conjugated mycotoxins are not sufficiently volatile for GC-MS analysis and so LC is widely used, with fluorescence or more commonly MS. ELISA methods can also be used directly provided cross-reactivity with free mycotoxins is checked, or it is used to estimate the total mycotoxin with its analogues that can indeed be useful for overall risk assessments. In general, immunoaffinity type tests are only used as screening techniques or for clean-up prior to LC. However, easy-to-use screening methods for both the parent toxin and the extractable conjugated forms should be further developed. The approaches described have relative advantages and disadvantages, and are complementary. Indirect methods cannot discriminate between different conjugates of the same toxin, and a lack of standards prevents calculation of the hydrolysis efficiency. Direct methods can identify specific conjugates but may not account for all of the bound toxins. Synthesis and (commercial) availability of reference standards are a prerequisite for accurate quantitation of masked mycotoxins. Furthermore, efforts should be put in the identification of unknown masked mycotoxin metabolites.

Available data are too limited to perform a quantitative hazard characterisation of masked mycotoxins. Dose response assessment and the establishment of e.g. a NOAEL is at present not possible. Masked mycotoxins can exhibit similar toxicity as their parent toxin as they eventually follow the same metabolic pathway. E.g. Z14G and ZEN are likely to show the same estrogenic effects in mammals, although such a study has not been carried out. Masked mycotoxins might also be less toxic than their parent compounds, if the hydrolysis of glucosides during digestion is incomplete. Finally, masked mycotoxins might also be more toxic than their parent compounds, e.g. when they are more bioavailable.

Currently it is impossible to perform a proper risk assessment for masked mycotoxins in food, due to the lack of data on exposure and toxic properties. Various forms of toxins clearly contribute to the toxicity of a given food and should be taken into consideration in setting future maximum residue limits. There is a clear need for more toxicological studies, preferably comparing the masked mycotoxin with its parent. Another possibility might be to use contaminated food plant commodities containing parent and masked mycotoxins and perform comparison toxicity studies with the pure mycotoxin. We strongly recommend further investigation of masked mycotoxins, in particular their occurrence, exposure and toxicity.

## Abbreviations

D3G: deoxynivalenol-3-β-D-glucopyranoside
DON: deoxynivalenol
FB_1_: fumonisin B_1_
FHB: Fusarium head blight
GSH: glutathione
GST: glutathione-*S*-transferase
HOAc: acetic acid
MeOH: methanol
OTA: ochratoxin A
UGT: UDP-glucosyltransferase
α-ZEL: α-zearalenol
α-ZELG: α-zearalenol-14-β-D-glucopyranoside
β-ZEL: β-zearalenol
β-ZELG: β-zearalenol-14-β-D-glucopyranoside
Z14G: zearalenone-14-β-d-glucopyranoside
Z14S: zearalenone-14-sulfate
ZEN: zearalenone
